# Investigation and contact tracing of the first cases of COVID-19 in Mozambique, 2020

**DOI:** 10.11604/pamj.2022.41.302.26979

**Published:** 2022-04-14

**Authors:** Judite Monteiro Braga, Auria Ribeiro Banze, Liliana Dengo-Baloi, Virginia Lara Evaristo, Erika Valeska Rossetto, Cynthia Semá Baltazar

**Affiliations:** 1 Instituto Nacional de Saúde, Marracuene, Mozambique,; 2 MassGenics, Assigned to Mozambique Centers for Disease Control and Prevention, Maputo, Mozambique

**Keywords:** SARS-CoV-2, contact, tracing, epidemiology, Mozambique

## Abstract

**Introduction:**

contact tracing is an important strategy to interrupt the spread of infectious disease and prevent new cases. After the confirmation of the first positive case of COVID-19 in Mozambique on March 22, 2020, case investigation and contact tracing were immediately initiated, which included clinical and laboratory monitoring of cases and contacts throughout the quarantine period. We aim to describe the methodology and impact of early investigation and contact tracing.

**Methods:**

in the context of implementation of the national COVID-19 preparedness and response plan, guidelines and forms for contact tracing were adapted from the existing World Health Organization (WHO) and The Centers for Disease Control and Prevention (CDC) guidelines. The case definition used was “patient with travel or residency history in a country reporting local transmission of COVID-19 during the 14 days prior to the onset of symptoms”. The cases interviews were face to face and contacts were followed up daily by phone calls for 14 consecutive days: using a structured questionnaire. Data were entered in an electronic Excel database. We collected samples for diagnosis of those who developed symptoms and provided quarantine follow up.

**Results:**

a total of 8 cases were confirmed, of which 6 (75%) were male. The average age of the cases was 51, median 44 (range: 31 to 80) years old. The majority of cases presented common symptoms of COVID-19, including headaches (50%), cough (37.5%), and fever (25%). Our case series included the country´s index case, two close positive contacts, and 5 additional cases that were not epidemiologically linked to the others and identified by the COVID-19 national surveillance system. All of them were identified in Maputo City from March 22 to March 28. Cases had a total of 123 contacts and all of them were tracked; 79 were contacts of the first case. From all the contacts in follow up, two had laboratory confirmed COVID-19. All cases and contacts were quarantined and none of them developed severe symptoms or required hospitalization.

**Conclusion:**

timely case identification and systematic contact tracing can be effective in breaking the chain of COVID-19 transmission when there is strong collaboration between epidemiological, laboratory surveillance and case management.

## Introduction

In 2020, a novel severe acute respiratory syndrome coronavirus 2 (SARS-CoV-2) emerged from Wuhan, China. The outbreak from SARS-CoV-2 was declared a public health emergency of international concern on January 30, 2020, and the disease spread rapidly in China and globally [1].

There are many aspects of the virus that are still unknown, including the clinical spectrum of disease, the severity and transmissibility. However, the most common symptoms of coronavirus disease (COVID-19) include fever, cough, and fatigue; whereas less common include production of sputum, headache, hemoptysis, and diarrhea [2]. The case fatality rate is not clearly established, although preliminary data suggests that it is less than 10% [2,3]. In response to the COVID-19 pandemic, the Mozambican Ministry of Health (MoH) developed a national preparedness and response plan that outlines strategic components for the management of the public health response to COVID-19 and outlines priority actions to take in the event of community transmission. This plan highlighted the need to conduct timely epidemiological investigation and contact tracing of positive cases.

Contact tracing is an important strategy to interrupt the spread of COVID-19, prevent new cases and control further transmission of the disease. The steps of this process include: 1) Identification of persons (contacts) exposed to confirmed cases; 2) listing of contacts, and; 3) monitoring persons regularly for symptoms and testing when in presence of signs of infection [4]. After the confirmation of the first positive case of COVID-19 in Mozambique on March 22, 2020, contact tracing was initiated immediately to rapidly track case contacts, collect samples for diagnosis of those who develop symptoms and provide quarantine to prevent the spread of the epidemic.

## Methods

During phase 1 of the COVID-19 national preparedness and response plan, during which no cases had been identified in the country, guidelines and forms for contact tracing were adapted from existing World Health Organization (WHO) and Centers for Disease Control and Prevention (CDC) materials. After the confirmation of the first case, the National Institute of Health (INS) activated a hotline for case reporting and four field epidemiology residents interviewed contacts using a structured questionnaire. Data from the interviews and laboratory testing were entered into an electronic database - Microsoft (Excel). Once the diagnosis of COVID-19 infection of the case was confirmed, contacts were traced immediately. The guidelines of WHO and CDC, recommends contacts to be identified by asking about the case´s activities and close contacts from 2 days before to 14 days after symptom onset [5].

A close contact was defined as an individual who had been exposed to a confirmed case of COVID-19 during the period of 2 days before the onset of symptoms of the confirmed case to 14 days after the onset of symptoms; exposure was defined as direct face-to-face contact within 1 meter of a confirmed case or equivalent prolonged (>15 min) exposure, including the following types of interactions: 1) Handshakes, kisses, hugs, or similar physical contact; 2) simultaneous/shared use of household and personal items; 3) sharing a dormitory/room; 4) sharing the interior space of a home at a distance of less than 1 meter and for more than 15 minutes; 5) providing direct care to a confirmed case without the use of personal protective equipment.

For the first confirmed case, we also implemented active follow up of low-risk contacts that we considered as indirect contacts. An indirect contact was defined as an individual who had a short (<15 min) period of contact with a confirmed case at a distance >1 m (1.5 - 2 m) in public settings, or an individual who had contact with the case in private settings that did not match the high risk of exposure definition. During the investigation, each COVID-19 positive case provided a list of contacts. Contacts were organized in a line list and demographic data, the relationship and date of last contact with the case, and the phone number and address of the contact were collected.

Based on the line list, individual forms were completed for each contact. The form was used to collect demographic data, the phone number, address of the contact and assessment of twelve symptoms: fever (>38°C), muscle aches, joint pain, weakness, nausea, vomiting, diarrhea, abdominal pain, headache, sore throat, shortness of breath, cough, and runny nose. The calls were conducted by Field Epidemiology and Laboratory Training Program (FELTP) residents and supervised by graduates; and a team rotation was established to work 7 days per week. To facilitate team rotation while maintaining a consistent standard of work, standard operating procedures were developed. During the call, the tracers used a script to introduce themselves, the monitoring objective, explain the period of symptom monitoring, provide information about prevention and control measures such as staying home during a 14-day period after their last contact with a confirmed case and to avoid contact with other persons living in the same household (or wear a surgical mask). The call ended with a reminder that the team would contact the person on the following day. If a contact reported symptoms, the FELTP resident was to notify the investigation team to determine if the contact met the COVID-19 case definition and therefore whether a sample needed to be collected. Data were entered and analyzed using Microsoft Excel®. Each form was considered as a unique contact record.

## Results

The first COVID-19 case was confirmed on March 22, 2020. The case was an 80-year-old man who travelled with a history of travel. He was tested for being listed as a positive case contact for an event he attended during the travel. The contact tracing team identified 79 contacts, classifying 30 as close contacts and 49 as indirect contacts. Among the close contacts were case 3 and case 8. Of the 30 close contacts of the case, six developed symptoms. Of the 49 contacts classified as indirect contacts, 14 reported symptoms (Table 1). All symptomatic contacts specimens were tested by real-time Reverse-Transcription Polymerase Chain Reaction (RT-PCR) at the Virology Laboratory at the Mozambican National Institute of Health. Aside from case 3 and case 8, no other contacts tested positive for COVID-19. The complete follow up of all contacts ended on April 11, 2020. Five additional confirmed cases were identified through March 28, 2020. All of them were quarantined at home and they developed mild symptoms such as sneezing, tiredness, and malaise.

**Table 1:** summary of characteristics and clinical features of the initial COVID-19 cases in Mozambique, 2020

	Case 1	Case 2	Case 3	Case 4	Case 5	Case 6	Case 7	Case 8
Age (years)	80	31	77	38	40	47	35	67
Sex	M	F	F	M	M	M	M	M
Date of symptom onset	NI	16/03/20	NI	NI	12/03/20	19/03/20	09/03/20	21/03/20
Date of case confirmation	22/03/20	23/03/20	24/03/20	24/03/20	25/03/20	25/03/20	24/03/20	28/03/20
Date of onset of symptoms'	24/03/20	-	26/03/20	-	-	-	-	-
Presenting symptoms at the date of confirmation of infection	Yes	Yes	Yes	No	Yes	Yes	No	Yes
Fever	No	No	Yes	No	No	No	No	No
Cough	No	Yes	No	No	No	Yes	No	Yes
Sputum	Yes	No	No	No	No	No	No	No
Difficulty breathing	No	No	No	No	Yes	No	No	No
Headache	No	Yes	No	No	Yes	Yes	No	No
Muscle soreness	No	No	Yes	No	No	No	No	No
Fatigue	No	Yes	No	No	No	No	No	Yes
Number of close contacts	30	9	30	8	4	3	3	17
Number of indirect contacts	49	-	-	-	-	-	-	-
Total number of contacts	79	9	44	8	4	3	3	17
Number of contacts tested	26	3	2	2	3	1	1	15
Number of contacts that developed symptoms	22	2	-	-	2	1	-	4

NI: no information; M: male; F: female

Case 2 was a 32-year-old female of Dutch nationality, who participated in a dance festival in Johannesburg, South Africa, from March 12 to March 15, 2020. She traveled to Maputo on March 16, 2020, on the flight, she didn't sit next to any passengers and developed fever, chills, fatigue, cough, muscle aches, body pain and sore throat on the same day. Infection with SARS-CoV-2 was confirmed on March 23, 2020. Epidemiological investigation identified one contact evaluated as low risk of infection. An additional eight contacts - all Mozambican nationals - who attended in the same festival in South Africa were followed up. During the follow up, one contact developed symptoms (sore throat, cough, headache, general weakness, sore throat, and abdominal pain). A sample was collected and tested negative by PCR.

Case 4 was a 38-year-old male, Mozambican nationality, who traveled in Europe (Portugal, Austria, and Switzerland) for 21 days. From March 11 to March 15, 2020, he and his family started quarantine and social distancing in Lisbon and returned to Mozambique on March 15, 2020. He remained in quarantine after his arrival in Mozambique. This case was initially diagnosed by a private laboratory on March 16; the results were available on March 24. He was asymptomatic. Contact tracing of this case identified nine contacts, and none had symptoms during the 14-day follow up.

Case 5 was a 40-year-old male, Mozambican nationality, who traveled from London to Maputo with a layover in Johannesburg on March 7 and arrival in Mozambique on March 8, 2020, he developed difficulty breathing and a headache. The case was first tested at a private laboratory on March 16, from which results were available on March 25. The Mozambican government was notified of the case by the testing private laboratory on that date. A total of four close contacts were identified and followed up for 14 days. During this period, two contacts developed symptoms, samples were collected, and tested negative by PCR.

Case 6 was a 47-year-old male, British. The onset of symptoms was on March 19, 2020, after returning to England from France on March 12. He developed body pain, muscle aches, joint pain, fever, nausea, and shortness of breath 2 days later. After 14 days of follow up he continued to experience headache and body pain, muscle aches, joint pain and tested positive by PCR. He had 3 contacts, including 1 adult who tested negative and 2 children who not tested did not develop any symptoms.

Case 7 was a 35-year-old male. This case was tested positive in a private laboratory on March 16, 2020, and the results were available on March 24, 2020. The Mozambican government was notified the same day the result was available by the testing private laboratory. The case was asymptomatic during the 14-day quarantine. The investigation identified three contacts, all of whom were asymptomatic during the 14-day of follow up.

Case 8 was a 67-year-old male and a close contact of first confirmed case. The epidemiological investigation team found that the last contact of case 8 with case 1 occurred on March 18, 2020. Three days later, symptoms of cough and malaise began. Samples were collected on March 26 and a PCR positive result was received on March 28. The case was asymptomatic during the 14-day follow up. This case had 8 close contacts, who all tested negative and who were asymptomatic during the follow up period. No health care workers (HCWs) that contacted cases or conducted epidemiological investigation, or the contact tracers developed symptoms or tested positive for SARS-CoV-2.

## Discussion

We found our approach; to implement contact tracing of the first cases of COVID-19 in Mozambique; to be highly effective in controlling the spread of COVID-19. However, this activity was highly demanding in term of human resources of the public health system. Our contact tracing and monitoring presented several challenges. Some contacts noted symptoms that were not related to the COVID-19 disease and others we were unable to trace (e.g. did not answer the phone). Additionally, not all passengers of the flights taken by cases who traveled internationally were interviewed and tested, however, we believe that the contacts most at risk were satisfactorily identified.

Initially, we did not strictly follow the definition of a contact of a COVID-19 case as any person who had contact with a COVID-19 case within a period ranging from 48 hours before the onset of symptoms of the case to 14 days after the onset of symptoms. Because of a delay in the receipt of results of 2 cases that were tested by a private laboratory, the contact tracing was extended more than 14 days after sample collection. A symptomatic case is another challenge for the contact tracing of COVID-19 cases. When an individual received a diagnosis of COVID-19, identification of his close contacts should have included those he was in contact with in the days prior to the onset of symptoms. However, asymptomatic cases may never seek medical attention, making it harder to trace chains of transmission.

The number of contacts per case ranged from 3 to 79, indicating that some contacts may not have met the inclusion criteria, thereby contributing to unnecessary use of resources. We collected data using a paper-based reporting system. Real-time data capture devices (such smartphone or tablets) may have improved the quality and increased the speed of the reporting system to guide associated interventions. Supervision and monitoring of contact tracing activities should be conducted on a daily basis to ensure consistency and effectiveness. Laboratory support is essential to the success of early detection of new cases.

## Conclusion

We described the implementation of contact tracing, monitoring and the first eight confirmed cases of SARS-CoV-2 in Mozambique. Effective real-time mobile-based contact tracing and monitoring could facilitate more timely data collection and analysis, and thus improve access to surveillance data to inform the national response strategy. We recommend additional analysis of surveillance data to build knowledge on the infectious period, modes of transmission, reproduction numbers, and effectiveness of preventive measures and case management in Mozambique.
